# Association of Outdoor Relative Humidity and Temperature on In-Hospital Cardiac Arrest Prognosis

**DOI:** 10.5334/gh.1266

**Published:** 2023-09-28

**Authors:** Taline Lazzarin, Edson Luiz Fávero Junior, Caroline Casagrande Delai, Victor Rocha Pinheiro, Raquel Simões Ballarin, Felipe Antonio Rischini, Bertha Furlan Polegato, Paula Schmidt Azevedo, Sergio Alberto Rupp de Paiva, Leonardo Zornoff, Antônio Ribeiro da Cunha, Adriana Polachini do Valle, Marcos Ferreira Minicucci

**Affiliations:** 1 São Paulo State University (Unesp), Medical School, Internal Medicine Department, Botucatu, Brazil; 2 Faculty of Agronomical Sciences, São Paulo State University (Unesp), Botucatu, Brazil

**Keywords:** Humidity, Temperature, cardiac arrest, mortality, return of spontaneous circulation

Environmental risk factors are a public health concern, and the literature on the health effects of climate variations is growing [1]. Climate factors such as air temperature (T; °C) and relative humidity (RH; %) have been linked to cardiovascular conditions such as myocardial infarction [2], sudden death [3], and cardiovascular mortality [4,5]. Cardiovascular conditions are the most often cause of cardiac arrest (CA) [6], so climate factors could also influence CA outcomes. Emerging evidence demonstrates the association between climate conditions and out-of-hospital cardiac arrest [7,8]. These results may be due to the interference of climate variability on outcomes affected by stress on the cardiovascular system by increased blood pressure, heart rate, increased blood viscosity, coagulability, and decreased systemic perfusion [9].

However, the relationship between climate influence and in-hospital cardiac arrest (IHCA) has not yet been evaluated. Thus, this study aimed to evaluate the association between outdoor air temperature, relative humidity, and IHCA outcomes.

This study was a subanalysis of an unpublished retrospective cohort that evaluated the risk factors of IHCA and was approved by the ethics committee of the Botucatu Medical School (56979721.9.0000.5411). The inclusion criteria were patients over 18 years old with IHCA, attended by the Rapid Response Team (RRT) in Botucatu Medical School University Hospital wards from May 2018 to December 2021. Exclusion criteria were the presence of do-not-resuscitate orders or recurrent IHCA. Demographic, laboratory, and clinical information were collected from the registry for electronic medical records. After that, data were complemented with air temperature (T; °C) and relative humidity (RH; %) on the day of diagnosis. Hospital wards were not climatized. Those data were collected in a nearby meteorology station (Faculty of Agronomical Sciences – Unesp, São Paulo State University, Botucatu, Brazil).

The RRT is a team triggered to the bedside to evaluate patients with signs of clinical deterioration, including CA, in our hospital. The outcomes of no-return of spontaneous circulation (no-ROSC) and in-hospital mortality were evaluated. We defined no-ROSC as the absence of ROSC or ROSC for less than 20 minutes. The statistical analyses were performed with SigmaPlot software for Windows v12.0 (Systat Software Inc., San Jose, CA, USA). Data are expressed as the percentage, mean ± standard deviation, and medians with 25th and 75th percentiles. Comparisons between two groups for continuous variables were performed using Student’s t-test or the Mann–Whitney test. Comparisons between two groups for categorical variables were made using the c2 or Fisher’s exact test. We constructed the multivariable logistic regression model for no-ROSC and the penalized likelihood with Firth logistic regression method for in-hospital mortality (due to the higher mortality rate in our sample). For both outcomes, the climate variables were adjusted by clinically relevant variables defined by the literature (epinephrine administration, initial shockable rhythm, time of CPR, gender, and age) or by variables that exhibited significant differences in the univariate analysis. The significance level was 5%.

In total, 407 patients were evaluated. However, 25 patients were excluded, 17 with do-not-resuscitate orders and 8 with recurrent IHCA. The mean age was 65.4 ± 14.8 years, 53.9% were male, and 91.1% of initial cardiac arrest rhythms were non-shockable. The no-ROSC rate was 56.8%, and in-hospital mortality was 94.2%. According to ROSC, the demographic, clinical, laboratory, and climate data are shown in Table 1. In this analysis, the only statistical factors related to ROSC were age and cardiopulmonary resuscitation (CPR) duration. However, in the multivariable logistic regression, the medium relative humidity (RH) was associated with no-ROSC when adjusted by age, gender, initial shockable rhythm, duration of CPR, and epinephrine administration [Odds ratio (OR) = 1.020; CI 95% = 1.003–1.038; p = 0.023], for each increase of 1g/kg of medium RH, there is an increase of 2% in the chance of no-ROSC. Also, the maximum RH was associated with no-ROSC when adjusted by the same factors [OR = 1.023; CI 95% = 1.003–1.044; p = 0.025]; for each increase of 1g/kg of maximum RH, there is an increase of 2.3% in the chance of no-ROSC. The other climatic parameters were not associated with no-ROSC. In multivariable logistic regression, there was no association between RH or temperature and in-hospital mortality. In addition, we performed a sensitivity analysis excluding patients admitted from March 2020 onwards to evaluate the possible impact of the COVID-19 pandemic on changes in local air pollution levels. Only 180 patients were included in this analysis, and we adjusted the models for the same factors. The association we observed with RH and no-ROSC with all the cohorts was lost with these 180 patients. For the association between medium RH and no-ROSC [OR = 1.014; CI 95% = 0.988–1.042; p = 0.294], and for maximum RH and no-ROSC [OR = 1.031; CI 95% = 0.995–1.068; p = 0.094]. This fact could be due to a reduction in the sample size.

**Table 1:** Baseline characteristics, laboratory and climatic data of 382 patients with in-hospital cardiac arrest according to return of spontaneous circulation (ROSC) and mortality.


VARIABLE	ROSC		*P* VALUE	MORTALITY		*P* VALUE
	
	Yes (165)	No (217)		Yes (365)	No (22)	

**Age (years)**	65.0 (55.0–73.0)	69.0 (58.0–78.0)	0.007	67.0 (57.0–77.0)	53.0 (43.5–68.5)	<0.001

**Male gender, n (%)**	86 (52.1)	120 (55.3)	0.607	167 (45.8)	12 (54.5)	0.560

**Initial rhythm, n (%)**						

**Shockable**	18 (10.9)	16 (7.4)	0.307	32 (8.8)	2 (9.1)	0.737

**Non-shockable**	147 (89.1)	201 (92.6)		333 (91.2)	20 (90.9)	

**Duration of CPR (min)**	14.0 (7.0–20.0)	30.0 (20.0–35.0)	< 0.001	24.0 (15.0–32.0)	6.0 (2.0–12.5)	<0.001

**Medical history, n (%)**						

**Arterial hypertension**	100 (60.6)	143 (65.9)	0.338	238 (65.2)	8 (36.4)	0.012

**Diabetes**	109 (66.1)	138 (63.6)	0.695	132 (36.2)	5 (22.7)	0.294

**Epinephrine, n (%)**	161 (97.6)	216 (99.5)	0.223	362 (99.2)	20 (90.9)	0.018

**Amiodarone, n (%)**	146 (88.5)	199 (91.7)	0.379	35 (9.6)	2 (9.1)	0.767

**Minimum Temperature (°C)**	17.0 (14.5–19.0)	16.7 (14.2–19.0)	0.948	16.8 (14.3–19.0)	16.4 (14.8–18.8)	0.999

**Medium Temperature (°C)**	21.1 (18.6–23.4)	20.8 (18.9–23.1)	0.683	20.9 (18.9–23.1)	21.6 (18.8–23.5)	0.771

**Maximum Temperature (°C)**	26.7 (23.4–29.7)	26.6 (23.9–29.3)	0.850	26.6 (23.9–29.4)	27.5 (23.3–30.7)	0.501

**Minimun RH (g/kg)**	41.8 (32.6–54.7)	43.3 (35.0–57.9)	0.189	42.9 (34.7–57.4)	42.2 (31.3–53.9)	0.456

**Medium RH (g/kg)**	66.4 (54.9–76.4)	68.2 (60.2–78.5)	0.067	67.3 (56.8–77.9)	69.3 (55.8–76.2)	0.638

**Maximum RH (g/kg)**	85.1 (77.7–91.5)	87.2 (80.5–92.7)	0.055	86.2 (79.1–92.0)	88.5 (80.3–92.5)	0.417

**Hemoglobin (g/dL)**	12.0 (± 2.5)	11.8 (± 2.5)	0.552	11.9 (± 2.6)	12.2 (± 1.9)	0.580

**Hematocrit (%)**	36.5 (± 7.4)	36.2 (± 7.4)	0.727	36.3 (± 7.5)	36.9 (± 5.2)	0.713

**Urea (mg/dL)**	55.0 (36.0–95.0)	58.5 (36.5–98.0)	0.231	57.0 (38.0–96.8)	27.5 (22.3–51.3)	<0.001

**Creatinine (mg/dL)**	1.0 (0.7–1.7)	1.1 (0.7–2.0)	0.426	1.1 (0.8–1.9)	0.7 (0.6–1.3)	0.005

**Sodium (mmol/L)**	136 (131–139)	136 (133–139)	0.162	136 (133–139)	135 (131–137)	0.136

**Potassium (mmol/L)**	4.3 (3.9–4.9)	4.4 (3.9–4.9)	0.662	4.3 (3.9–4.9)	4.3 (4.0–4.9)	0.947


Data are expressed as the mean ± SD, median (25–75%) or percentage. CPR = cardiopulmonary resuscitation; ROSC = return of spontaneous circulation; RH = relative humidity.

Regarding the changes in the environment, the effects of extreme weather on human health have been studied in recent years. The temperature-mortality curves were generally U-shaped, with higher risks in extreme temperatures [4]. The temperature could modulate blood pressure, heart rate, blood viscosity, coagulability, and systemic perfusion [9]. The effects of RH are less clear. However, some studies suggest that high RH could reduce the body’s efficiency at transporting away metabolic heat, while low humidity could lead to dehydration [4]. Thus, our study highlights the importance of humidity in IHCA prognosis. We should consider some limitations of our study, such as the retrospective design and the use of RH instead of absolute humidity, which is a better indicator of indoor humidity [10]. In conclusion, there is an association between RH and ROSC, but not mortality after IHCA.
